# Evaluation and Monitoring of *Mycobacterium leprae* Transmission in Household Contacts of Patients with Hansen's Disease in Colombia

**DOI:** 10.1371/journal.pntd.0005325

**Published:** 2017-01-23

**Authors:** Marcela Romero-Montoya, Juan Camilo Beltran-Alzate, Nora Cardona-Castro

**Affiliations:** 1 Instituto Colombiano de Medicina Tropical–Universidad CES. Department of Microbiology, Sabaneta, Antioquia, Colombia; 2 Facultad de Medicina Universidad CES. Medellín, Colombia; Fondation Raoul Follereau, FRANCE

## Abstract

Leprosy in Colombia is in a stage of post elimination—since 1997, prevalence of the disease is less than 1/10000. However, the incidence of leprosy has remained stable, with 400–500 new cases reported annually, with MB leprosy representing 70% of these case and 10% having grade 2 disability. Thus, leprosy transmission is still occurring, and household contacts (HHCs) of leprosy patients are a population at high risk of contracting and suffering from the effects of the disease during their lifetime. We performed a cross-sectional study with the aim of evaluating leprosy transmission within Family Groups (FGs) from four Colombian departments: Antioquia, Bolívar, Córdoba and Sucre. This study included 159 FGs formed by 543 HHCs; 45 FGs were monitored twice, first in 2003 and again in 2012. Migration, forced displacement by violence, loss of contact with the health center and the lack of an agreement to participate in the second monitoring were the primary reasons not all FGs were tested a second time. In each HHC, a clinical examination was performed, epidemiological data recorded, the bacillary index determined, DNA was isolated for *M*. *leprae* detection by nested PCR and IgM anti-phenolic glycolipid-I (PGL-I) titers were inspected. Further, DNA from *M*. *leprae* isolates were typed and compared among FGs. Twenty-two (4.1%) of the 543 HHCs had IgM anti-PGL-I positive antibody titers, indicating infection. Nasal swabs (NS) taken from 113 HHCs were tested by RLEP PCR; 18 (16%) were positive for *M*. *leprae* DNA and two new leprosy cases were detected among the HHCs. Of the confirmed HHCs with leprosy, it was possible to genotype the bacterial strains from both the index case and their HHCs. We found that the genotype of these two strains agreed at 9 markers, showing the individuals to be infected by the same strain, indicating familiar transmission. HHCs of leprosy patients not only are a high-risk population for *M*. *leprae* infection, they can act as *M*. *leprae* carriers and therefore serve as sources for transmission and infection. Our results confirm familiar leprosy transmission and suggest that follow-up of HHCs is a good strategy for early diagnosis of leprosy and to monitor its transmission.

## Author Summary

Leprosy in Colombia is considered in a post-elimination stage, as prevalence of the disease is less than 1/10000 since 1997. However, leprosy transmission is still common with 400 to 500 new cases reported each year—70% of them multibacillary (MB) and 10% with grade 2 disability, demonstrating late diagnosis. HHCs of leprosy patients are a population at risk for infection by *M*. *leprae* and the subsequent development of leprosy. However, the *M*. *leprae* incubation period is long, and measures to follow-up with this population are difficult and not included in leprosy control programs in Colombia. We performed this survey with the aim to evaluate leprosy transmission in family groups of leprosy patients from four Colombian departments: Antioquia, Bolívar, Córdoba and Sucre. Volunteers (n = 713), 170 (24%) leprosy patients and 543 (76%) HHCs belonging to 159 family groups (FG) were included after informed consent was given. Of these volunteers, 225 (31.5%) were monitored two times: 45 leprosy patients and 180 HHCs. Volunteers were given a clinical examination and epidemiological data was recorded. Skin biopsies, nasal swabs and slit skin samples from patients were taken for bacillary index determination and *Mycobacterium leprae* genotyping. Nasal swabs and slit skin samples from HHCs were tested by nested PCR. Additionally, serum samples were tested for IgM anti-phenolic glycolipid-I (PGL-I) titers. Twenty-two (4.1%) of the 543 HHCs had IgM anti-PGL-I positive antibody titers, indicating infection. PCR of DNA isolated from nasal swabs was positive for *M*. *leprae* in 18 (16%) HHCs, suggesting the presence of carriers. Two new leprosy cases were detected among the HHCs. We found three leprosy cases in one family group, two of them multibacillary. The *M*. *leprae* genotype of these two strains agreed at 9 markers, showing these individuals are infected by the same *M*. *leprae* strain, indicative of familiar transmission. Our results confirm that leprosy transmission is active in a country where leprosy is in post-elimination stage.

## Introduction

Leprosy, also known as Hansen's disease, is an infectious and chronic disease caused by *Mycobacterium leprae* [1]. The mode of transmission of *M*. *leprae* has not yet been demonstrated, although entry through the nasal passages is a commonly accepted potential mechanism [2]. While humans are the main reservoir of *M*. *leprae*, nine-banded armadillos are also known to serve as reservoirs of this bacterium [2]. It is estimated that about 2 million people worldwide have some type of disability due to leprosy [3]. While multidrug therapy (MDT) has been highly effective in treating leprosy infections, treating nerve damage that results from the disease has proven more difficult [3].

In Colombia, the detection of new leprosy cases decreased in 2009 and 2010. However, the number of new cases remained stable in 2011 (434 cases), 2012 (364 cases), 2013 (433 cases), 2014 (370), and again in 2015 (349) [4, 5]. These data suggest that the transmission of leprosy in Colombia continues despite the country classified as being in a period of post-elimination. It has been observed that the global decrease in leprosy prevalence has not been accompanied by a decrease in the incidence of the disease [6]. The late diagnosis of leprosy in Colombia is evident by the proportion of multibacillary (MB) to paucibacillary (PB) leprosy cases of 70/30, with 10% of MB patients having grade 2 disability. Thus, the prevention of transmission has not been achieved despite the implementation of MDT programs. Further complicating matters is the under-reporting of the disease [6]. Prominent reasons why the incidence of the disease continues in endemic countries appears to be the presence of reservoirs within infected populations—sub-clinical leprosy or non-human environmental sources that have not been detected [7–9].

In comparison with the general population, household contacts (HHCs) of leprosy patients are a population at high risk of contracting the disease and suffering the effects of *M*. *leprae* infection during their lifetimes. Studies have demonstrated that most new leprosy patients have had contact with another patient [10,11]. Due to the long and imprecise incubation period of leprosy, it cannot be determined which HHC will ultimately develop leprosy. Further, Colombian health programs do not regularly monitor HHCs of leprosy patients. Using enzyme-linked immunosorbent assays (ELISA) to determine *M*. *leprae* infection, the phenolic glycolipid-1 (PGL-I) has been found to be specific to *M*.*leprae* [10]. While MB patients generate antibodies against PGL-I, PB patients do not. In HHCs of leprosy patients, detection of these antibodies may be indicative of infection but offer no protection against the disease [10, 12–14].

One form of protection for HHCs used in some countries is the Calmette-Guerin Bacillus (BCG) vaccine [15], recognized for its protection against *Mycobacterium tuberculosis* infection. The protective effect of the BCG vaccine to non-infected persons ranges from 10–80% [16], with the vaccine considered a stimulus to the immunologic reactivity of the HHCs of leprosy patients. It is possible that the combination of medication and BCG vaccine may facilitate elimination of *M*. *leprae* in the host (by increasing TNF-α and IL12 levels and activating macrophages), decrease relapse rates and shorten the positivity of the bacilloscopy [16,17].

Molecular tests have been developed that detect specific *M*. *leprae* nucleic acids with high sensitivity and specificity and are used to confirm the diagnosis of leprosy in PB patients and to detect the bacterium in asymptomatic HHCs [18,19]. Likewise, advances in the genotyping *of M*.*leprae* based on insertions, deletions, Single Nucleotide Polymorphisms (SNPs) and Short Tandem Repeats (STRs) have revolutionized our understanding of leprosy’s origins, its patterns of migration and propagation and the disease’s resistance to drug treatment [20]. These tools can be used to better understand areas where the disease is present and its means of transmission [18–21].

Leprosy control programs in Colombia include a clinical review of HHCs immediately after diagnosis of the index case has occurred [22]. This vigilance is important, but not sufficient because leprosy has a variable period of latency; clinical follow-ups for several years are necessary to detect the early stages of the disease. Additionally, a clinical exam is not a good tool to detect subclinical cases of the disease [23].

In the current study, we monitored leprosy transmission in HHCs of patients with leprosy. We examined clinical, bacteriologic, and immunologic changes in the HHCs. We also monitored genetic markers in the bacterium, which may improve early detection and improve knowledge about transmission of the disease, thereby avoiding late diagnosis and preventing permanent damage resulting from the disease.

## Materials and Methods

### Ethics statement

This study was approved as the minimal risk by the ethical committee of the Instituto Colombiano de Medicina Tropical–Universidad CES. An informed consent form was signed by patients, HHCs, and parents or tutors of children under 18 years of age.

### Description of the population and sample

A cross sectional survey was performed in the leprosy cases, and their HHCs, registered from 2003 to 2012 in the Colombian departments of Antioquia, Bolívar, Córdoba and Sucre. Leprosy patients and their HHCs were monitored once or twice by examining their epidemiological, clinical, bacteriologic, and IgM PGL-I antibody titer changes. The first monitoring was performed in 2003, the second in 2012. All volunteers, parents or tutors of children signed a consent form to participate in this survey.

For each index case (leprosy patient) and HHC (family member or any person that lived under the same roof with the index case for more than six months), a clinical record was filed which included medical and epidemiological data. Age, sex, the relationship with the index case, and detection of a BCG vaccination scar were recorded. Finally, clinical symptoms were recorded as well as data regarding the treatment stage according to each individual.

### Clinical exam

Each HHC was examined for signs and symptoms of leprosy. This included the detection of areas of hypoesthesia or anesthesia, thermic sensibility to cold and heat, palpation of the nerve trunks, presence of hypo- or hyper-pigmented lesions, unnoticed burns or wounds, nodules, atrophy, contractures, anomalous positions of the fingers, loss of muscular strength and an alteration of motion. A HHC was classified as symptomatic when he or she presented at least one of these symptoms.

The classification of leprosy was performed according to World Health Organization (WHO) recommendations. Patients classified with MB leprosy had a positive bacillary index (BI) and more than five skin lesions. Patients classified with PB had a negative BI and less than five skin lesions [6]. For the prescription of treatment, clinical classification by Ridley and Joplin [8] was also used.

### Detection of *M*. *leprae* Infection

#### Bacillary Index (BI) and Zielh Neelsen (ZN) stain

Slit skin smear (SSS) samples and nasal swabs (NS) were stained with ZN to test for acid-alcohol-resistant bacilli. ZN staining was performed with a steam emission of fuchsine for 10 minutes, discoloration with acid-alcohol for 3 minutes and coloration of contrast with methylene blue for 2 minutes.

#### ELISA to detect IgM anti-PGL-I antibodies

We tested for the presence of IgM anti-PGL-I using the PGL-I antigen according to the methodology described in [13,14,15].

#### DNA extraction from biopsy, slit skin samples and nasal swabs (NS)

Slit skin samples were obtained from earlobes, the margins of lesions and elbows by puncture with a sterile lancet. Nasal swabs and biopsies of HHCs with suspected Hansen’s disease lesions, as well as their index case, were stored in 70% ethanol until DNA extraction. DNA extraction was performed with the QIAGEN DNeasy Blood & Tissue Kit according to the manufacturer’s protocol.

#### Nested PCR to detect *M. leprae* DNA

Nested PCR was carried out to amplify repetitive elements (RLEP) specific to *M*. *leprae* using DNA obtained from the NS of the HHC. LP1-LP2 primers for PCR, and LP3-LP4 primers for nested PCR, were used and PCR was performed as in [19]. PCR cycling parameters were as follows: 95°C for 2 minutes, denaturation at 95°C for 30 seconds, annealing at 55°C for 45 seconds and elongation at 72°C for 30 seconds. PCR was conducted for 45 cycles. A final extension step of 72°C for 5 minutes was included. For nested PCR, the amplification product was diluted to 1:400. Conditions for each PCR remained constant.

Electrophoresis was performed in a 2.5% agarose gel in Tris–borate–EDTA buffer (1X TBE). Gels were visualized with ethidium bromide. The presence of *M*.*leprae* DNA was evidenced by the observation of a band of 129 bp for the direct PCR and 99 bp for the nested PCR. As amplification controls, we used a positive control (NHDP63 strain DNA) and a negative (no DNA) control.

#### Molecular typing and sequence

We used primers according to previously published sequences [19,21,24] to amplify regions of the *M*. *leprae* genome containing short tandem repeats (STR); of these, we used 12 variable number tandem repeats (VNTR) and 1 single nucleotide polymorphism (SNP) 7614 in *gyrA* gene (Table 1) from samples of lymph or biopsy of patients.

**Table 1:** Loci characteristics and primers used for *M*. *leprae* typing.

Primers	Sequence (5ʾ - 3ʾ)	Unit Repetition (pb)	Locus	Size (pb)	Access No^α^
**RP6-3**	CTA CTT GCG CGC CAC CGC CA CCG TCG CCA GGT TTT GCA GA	6	6–7	191	ML1505/+
**RP12-1**	AGT AGC TTC CAT CCC CTC AT GCG ACG AAA GCA TTT ACG GC	12	12–5	289	ML1182
**RP18-1**	GCT ATG GGC AGC CTG GGT AT AGC CGG TTA CCA AGA TGG CA	18	18–8	330	ML1334/+
**RP21-1**	TGT TGA AAT TTG GCG GCC AT TGC AAG GAG TGC TCA GCT AT	21	21–3	179	ML0058/-
**RP23-1**	CAG TCG CCC GGA TAC TGT TA TAA ATC CGC TCC CAA ATC TT	23	23–3	190	ML2469-ML2470
**RP27-1**	GTG CTG TGC CTG CCG TT TCC CCA AAG CCG CCG AAT CC	27	27–5	270	ML0568/+
**RP2-2**	GTG TTA CGC GGA ACC AGG CA CCA TCT GTT GGT ACT ACT GA	2	(AC)8a	124	ML1285/-
**RP2-3**	GAT GCG ACT ATC ACT CGC AC GCT GGT TTC CTT CTA GTC CC	2	(AC)8b	140	ML1824-ML1825
**RP3-2**	TCA CCA TCG ACG CTC CGG GT TCG GCC TGG TTG TCT GCC TT	3	(GGT)5	161	ML2159-ML2160
**RP2-4**	GCC TGG TGC CCG GAC AAT GC ACT GAT CTC GCC GGC GCT GT	2	(AC)9	140	ML1227-ML1228
**RP2-12**	TTA GCA GGA CGA TTG TAC AG ACC CGG AAT TCC TCC AAG	2	(AT)17	160	ML2183/-
**gyrA**	CCGTAGCCACGCTAAGTCA	-	-	158	SNP7614

PCR cycling parameters were as follows: 95°C for 2 minutes, denaturation at 95°C for 30 seconds, annealing at 65–55°C for 45 seconds and elongation at 72°C for 30 seconds. PCR was conducted for 45 cycles. A final extension step of 72°C for 5 minutes was added.

Electrophoresis was carried out in agarose gel 2.5% in 1X TBE buffer. Gels were visualized with ethidium bromide. The presence of DNA of *M*. *leprae* was evidenced through the observation of a band of different molecular weight according to the VNTR used.

Amplicon sequencing was performed at the Leprosy Research Center, National Institute of Infectious Diseases, Tokyo Japan, using an ABI 3130 Genetic Analyzer (Applied Biosystems).

### Data analysis

A descriptive and bivariate analysis of the data was performed using Statistical Package for the Social Sciences program (SPSS Inc, Chicago, IL) PASW Statistics 18. The odds ratio (95% CI) was calculated and a P value < 0.05 was considered significant.

## Results

### Patients and family groups (FGs)

A total of 159 FGs comprised of 713 individuals were included in this study: 170 leprosy patients (24%) and 543 HHCs (76%). A total of 225 individuals corresponding to 32% of the study population were monitored twice: 46 leprosy patients (20.4%) and 180 HHCs (44.8%). Table 2 describes the characteristics of the leprosy patients and their family groups.

**Table 2:** Characteristics of leprosy patients and household contacts (HHCs).

Department/Characteristic		Antioquia	Bolívar	Córdoba	Sucre	Total
#Family groups		44	73	23	19	159
#MB/#PB Patients		38MB/8PB	63MB/14PB	19MB/7PB	15MB/6PB	135MB/35PB
#Patients without HHC		5	3	4	1	13
Patients with one HHC		9	13	7	1	30
Patients with 2–5 HHC		28	34	12	8	82
Patients with 6–10 HHC		3	19	3	11	36
Patients with 11–19 HHC		1	4	0	0	5
# HHC		146	291	49	57	543
HHC <15 years old		31	96	11	11	149
Patients <15 years old		1	3	0	0	4
Family groups with more than one patient under treatment		1	2	1	1	5
#Family groups with at least one HHC IgM anti PGL-I positive titers	2003	6	20	5	6	37
	2012	3	11	8	6	28

Of the 170 leprosy patients, 135 (79.4%) were MB and 35 PB (20.6%). A higher frequency of leprosy was found in men than in women with a ratio of 1 woman per 3.4 men.

The average age of the patients was 53 years with a variation of 17.6 years. Half of the patients were over 51 years old with a variation of 28 years; the interquartile range (IQR) was 40–68.3. The minimum age was 5 years and the maximum age was 90 years.

In this study we found four leprosy patients undergoing treatment younger than 18 years of age: a 5 year old girl with MB leprosy who had an uncle as a family contact, a 9 year old boy with MB leprosy whose mother was in treatment for MB at the moment of his diagnosis and two young boys of 14 and 16 years old, both with MB leprosy. Primary school was the highest educational level achieved by 83 (48.8%) of the leprosy patients studied.

BCG scars were evident in 27 (15.9%) of the patients. 114 (67.1%) of patients did not have the scar and in 29 (17.1%) it was not possible determine if a vaccination was carried out. We found a statistically significant relationship between positive BCG scars and not having leprosy (p = 0.0001), OR: 0.131, IC (95%): 0.083–0.207. We did not observe a statistically significant relationship between receiving the vaccination and MB vs. PB leprosy (p = 0.2615), OR: 1.867, IC (95%) 0.619–5.627.

### Household contacts

Average age of the HHCs was 32 ± 20.3. Half of the HHCs were over 27 years old with a variation of 32 (IQR was of 15–47). The minimum age was 1 year and the maximum was 90 years. The 543 HHCs and the 170 leprosy patients belonged to 154 family groups. Table 2 shows the families characteristics.

#### BCG scar in HHCs

A BCG scar was evident in 326 (60%) of the HHCs, 181 (33.3%) were negative for a BCG scar and in 36 (6.6%) it was not possible to determine the presence of a BCG scar. We found a potential protective effect of the BCG scar and negative titers of IgM anti-PGL-I. However, this result is not conclusive as the p value only bordered on significance (*p = 0*.*05*).

#### Exposure time of the HHC

91.2% of the HHCs had exposure to the index case for at least two years. We did not observe a statistically significant relationship between the exposure time with the patient and the IgM anti-PGL-I titers (*p>0*.*05*), OR: 0.51, IC95%: 0.063–4.131.

#### ELISA IgM anti PGL-I

Twenty-two (4.1%) of the 543 HHCs had positive IgM anti-PGL-I antibody titers, 7 of them were negative for IgM anti-PGL-I antibodies at the first monitoring in 2003 but showed positive titers in 2012. The index cases of 21 HHCs (95.5%) with positive IgM anti- PGL-I titers were MB. In contrast, 1 (4.5%) HHC who had contact with a PB patient showed positive titers for IgM anti-PGL-I (p<0,05). 14 of the 22 HHCs (63%) are consanguineous with the index case in the first (parents and children) and second (sisters and brothers) degree. In one family, three HHCs showed positive titers for IgM anti-PGL-I. Four families had two HHCs with positive IgM anti-PGL-I titers.

#### Nasal swab (NS) RLEP PCR

NS of 113 HHCs were tested by RLEP PCR. Nasal swabs of 18 of 113 HHCs (16%), belonging to 12 FGs, tested positive for *M*. *leprae* DNA. This suggests that two FGs had two HHC carriers of *M*. *leprae* and an additional FG had three HHC carriers of *M*. *leprae*.

#### Confirmation of new leprosy cases in the HHCs

Two new cases of leprosy were detected in one FG where the grandfather is the index case. The first case is the 33 year-old son of the index case, MB with lepromatous leprosy (LL). The second case is the 5 year-old grandchild of the index case, PB with a diagnosis of indeterminate leprosy.

### *M*. *leprae* typing

*M*. *leprae* typing of the index case (in the FG where the two new cases were detected), and one of the new cases (a MB patient), confirmed familiar transmission. Table 3 shows the genotypes of both *M*.*leprae* isolates.

**Table 3:** Genotyping of *M*. *leprae* isolates of the index case and HHC new case.

Status. Parental Relationship	Age	BI	Clinical Manifestations	IgM anti PGL-I titers	Leprosy diagnosis	Genotyping											
						6–7	12–5	27–5	18–8	21–3	23–3	(AC)8a	(AC)8b	(GGT)5	(AC)9	(AT)17	GyrA
Patient DSSA562	65	2.4	Anesthesia, hypo/hyper pigmented macule, nodules, lepromas.	0.623 POS	MB-LL	NA*	5	3	3	2	2	11	7	4	NA	NA	C
HHC-Son. DSSA575	33	2.8	Nodules, lepromas, erythematous skin lesions, loss of sensitivity in hands and foot.	0.536 POS	MB-LL	6	5	3	3	2	2	10	7	4	8	14	C

BI: Bacillary Index. POS: positive. MB: multibacillary. PB: paucibacillary. LL: lepromatous Leprosy. IL: Indeterminate Leprosy. NA*: Non-amplification.

#### Study limitations

This study included 159 FGs, 45 of which were monitored twice, the first time in 2003 and the second in 2012. Migration, forced displacement by violence, loss of contact with the health center, and lack of an agreement to participate in the second monitoring were reasons not all FGs were tested twice.

## Discussion

This study describes leprosy transmission from index cases to their family groups in the Colombian departments of Antioquia, Bolívar, Córdoba and Sucre from 2003 to 2012. Clinical exams, bacillary index, RLEP PCR, IgM anti-PGL-I titers and *M*. *leprae* genotyping were performed to determine leprosy transmission.

Of the leprosy patients and HHCs monitored, it was possible to contact 225 of them (32%) a second time. Due to the lack of follow up from Hansen’s programs after treatment, it is difficult to contact patients and their families again.

We found a greater incidence of the disease in men (77.1%) compared to women, which coincides with other studies [25,26]. We observed no relationship between gender and the MB or PB status (p>0.05) [26].

Leprosy in Colombia is considered to be in post elimination phase [27]. However, the four children under 14 years of age that are currently undergoing treatment and the new case (a 5 year old) diagnosed during this study are important epidemiological reminders that should be considered indicators of the prevalence of the disease in the general population as well as a sign of ongoing transmission. Some studies suggest that the long incubation period of the disease affects children in the age range of 10–14 years old; nevertheless, the affected children between 1 and 9 years old likely reflect their early exposure to active cases of the disease [28,29] and/or to areas of transmission within communities [28].

The socio-economic status of the leprosy population was revealed during this study: 48.8% of the patients only had a primary school level of education and 27.1% did not receive any type of scholarly study, results in accordance with other reports [30]. One of the difficulties we encountered during the socio-epidemiologic survey was the lack of information from patients regarding their age, their knowledge of the disease, any previous MDT treatment, the number of supplied doses of MDT, complementary treatments, the date of diagnosis of the disease and their current treatment status. This reflects the patient’s lack of education regarding leprosy.

The BCG vaccine is known to protect against *Mycobacterium tuberculosis* infection; cross protection of the vaccine for *M*. *leprae* ranges from 10 to 80% [16]. In this study, the BGC vaccine showed a protective effect of 87% (OR: 0.13, IC95%: 0.08–0.21). However, to confirm this result, a follow up of the same population must be performed. Being a protective measure, we found a high percentage of leprosy patients that had not received the BCG vaccine, primarily due to the fact that leprosy patients in Colombia are not vaccinated at an early age—the majority of those vaccinated are in their adult years—or their access to the vaccine was limited or unavailable in the areas where they live [16]. Leprosy control programs in Brazil recommend the BCG vaccination to all the healthy persons who are in contact with leprosy patients [15]. In Colombia, BCG vaccination of HHCs has been established for their protection [31]. Our results show that 60% of HHCs had evidence of receiving the vaccine in the form of a scar while 33.3% did not, indicating that the Hansen’s disease programs of these departments do not implement revaccination to 100% of HHCs after diagnosis of the index case.

Use of the BCG vaccine is considered a stimulus for immunological reactivity, possibly due to the fact that the combination of MDT and the BCG vaccine may facilitate the elimination of *M*.*leprae* from the patient (increasing the TNF alpha, IL12 and activating macrophages), decreasing the rates of relapse and reducing the positivity of the BI [16,17].

Numerous studies have demonstrated that leprosy appears to have a relationship between the clinical outcome of the disease and a familiar relationship with a leprosy patient. Correa et al [30] found that the reports of family leprosy are of first and second grade consanguinity. The current study found that 495 (91.2%) of the HHCs have had an exposure time of years with the index case, while only 2 (0.4%) had occasional contact, suggesting that HHCs are exposed for a prolonged time to BI positive patients without a diagnosis.

The presence of PGL-I antibodies in the HHCs of patients has been widely studied. Nevertheless, few studies have performed long-term monitoring of HHCs [10]. The current study shows IgM anti-PGL-I in 4.1% of HHCs. However, we did not find a statistically significant relationship between the time of exposure of the HHC and positive IgM anti-PGL-I (p > 0.05). The positive IgM anti-PGL-I in non-symptomatic HHCs suggests infection without the disease; follow-up of these HHCs is needed to determine if these HHCs eventually develop the disease.

Klatser et al [32] found *M*. *leprae* DNA in nasal swabs in 7.8% of 1228 samples from an endemic population. In this study of 113 HHCs, 16% showed a positive PCR. These results suggest that HHCs may act as hosts of *M*.*leprae* and therefore could be a source of infection and transmission. Thus, it is necessary to perform periodic clinical examinations and complementary exams to diagnose the disease early in high-risk populations.

Leprosy detection in two symptomatic HHCs of the same family whose index case was an MB patient confirms the transmission of leprosy between family members, which has been considered a main mode for the propagation of the infection in BI positive patients without treatment [33]. The index case corresponds to the father and the HHC to the son. The genotype of these two strains agreed at 9 markers; two markers did not amplify and one marker did not agree between the two strains (AC8a), which is highly polymorphic. Genotype comparisons will allow monitoring of the circulating strains in the region in general, and in the affected homes in particular. However, it is necessary to take samples from the index case prior to treatment for *M*.*leprae* to allow comparisons with the new isolates from family cases or contacts.

Only a small minority of the human population develop leprosy because *M*. *leprae* infection is unlike the universal susceptibility to other members of the *Mycobacteriaceae* family. It’s accepted that the majority of the humans are immune to leprosy through an as yet defined mechanism [33]. That a small minority of persons who succumb to the disease are diagnosed late leads to the acquisition of disabilities that alter their familial, social and occupational environment. An early diagnosis that includes the correct monitoring of the index case and HHCs would assure cutting the chains of transmission in both the family and the community.

## Conclusions

Follow up of HHCs is a public health decision that can improve leprosy control. The presence of anti-PGL-I antibodies and *M*. *leprae* DNA in HHCs can suggest infection and source of infection and transmission of leprosy. The genotyping of *M*. *leprae* strains between family members allowed us to establish the source of transmission and make comparisons between the circulating *M*. *leprae* strains of a specific region.

Follow-up of HHCs using clinical exams to detect skin or peripheral nervous system symptoms of the disease, and the detection of infection using anti-PGL-I antibodies and *M*. *leprae* DNA immediately upon diagnosis of the index case may allow us to establish better methods to control the transmission of the infection.
